# Comparative analysis of mitogenomes among three species of grasshoppers (Orthoptera: Acridoidea: Gomphocerinae) and their phylogenetic implications

**DOI:** 10.7717/peerj.16550

**Published:** 2023-12-15

**Authors:** Li Wang, Jianyu Chen, Xiaobao Xue, Guoqing Qin, Yuanyi Gao, Kai Li, Yulong Zhang, Xin-Jiang Li

**Affiliations:** The Key Laboratory of Zoological Systematics and Application, School of Life Sciences, Institute of Life Sciences and Green Development, Hebei University, Baoding, China

**Keywords:** Gomphocerinae, Grasshopper, Mitogenome, Phylogenetics

## Abstract

Whole mitochondrial genomes have been widely used in phylogenetic analysis, population genetics and biogeography studies. This study sequenced and characterized three complete mitochondrial genomes (*Dasyhippus peipingensis*, *Myrmeleotettix palpalis*, *Aeropedellus prominemarginis*) and determined their phylogenetic position in Acrididae. The length of the mitochondrial genomes ranged from 15,621–15,629 bp and composed of 13 PCGs, 2 rRNA, 22 tRNA genes and an AT control region. The arrangement and structure of the mitochondrial genomes were similar to those of other invertebrates. Comparative genomics revealed that the three mitochondrial genomes were highly conserved in terms of gene size, structure, and codon usage, all PCGs were purified selections with an ATN start codon and a TAN stop codon. All tRNAs could be folded into the typical clover-leaf structure, except tRNA Ser (AGN) that lacked a dihydrouridine (DHU) arm. Phylogenetic analysis based on 13 PCGs of 34 Acrididae species and seven outgroup species revealed that differences in the shape of antennae within the family Acrididae should be given less weight as a taxonomic character for higher-level classification. Moreover, the divergence time estimates indicates that in Gomphocerinae, the species with clubbed antennae were formed within the nearest 18 Mya, and *Pacris xizangensis* is more ancient.

## Introduction

Insects originated about 479 Mya in the Early Ordovician, and a pair of antennae can be found in the evolutionary lineage, which have evolved into various shapes during the subsequent evolution (Misof et al., 2014). Antennae are important structures for receiving and transmitting information, sensing chemical odors, humidity, and temperature of the external environment, and playing an important role in finding hosts, mating, and defense (Lan, Xiang & Zhu, 2023). The shape of antennae, the number of segments, the location of antennae and sensilla on the surface of antennae vary with different insect species. Li, Ren & Wang (2013) studied the antennal sensilla types and distribution of six species in three genera in tribe Mylabrini on the preliminary determination of the interrelationship and taxonomic status in genus category. In Acrididae, the shape of antennae is variable, the external morphology of the antennae in most species is filiform (antennal flagellum with a circular or ovoid cross section and approximately equal diameter in cross section), while other grasshoppers are ensiform (antennal flagellum with some enlarged segments at the base and a triangular cross section) or club shaped (antennal flagellum with an enlarged end that resembles a club). There are quite different taxa with clubbed antennae within Acrididae, *e.g*., Egnatiinae, Eremogryllinae, Oedipodinae and Gomphocerinae *etc*. In addition, clubbed antennae do not occur exclusively in Acrididae. Similar antennal shapes are found in some closely related groups such as the Eumastacidae (*Myrmaleomastax* and *Pentaspinula*) and Tetrigidae (*Discotettix*), and in distantly related species such as most butterflies in Lepidoptera, Ascalaphidae in Neuroptera, and some Coleoptera.

Gomphocerinae, a subfamily of Acrididae (Orthoptera), has the type genus *Gomphocerus* Thunberg, 1815. Priority for family-group names based on *Gomphocerus* was established by Fieber in 1853, and the first use as Gomphocerinae was by Uvarov in 1966 (Cigliano et al., 2023). This subfamily is composed of 192 genera and 1,274 species (Song et al., 2018), it is one of the most diverse and species-rich taxa in Acrididae, with its main habitats being tundra and swamps, extreme deserts, and tropical rainforests (Otte, 1981). In this subfamily, the species are distinguished due to presence of stridulatory mechanism that produces reproductive sounds, located in the internal femur region (Jago, 1971). Many researchers followed the original definition of the subfamily, whereas it suggests that the stridulatory mechanism may be a characteristic originated from convergent evolution which emerged in different moments during the Gomphocerinae diversification or that originated in the Gomphocerinae ancestral and was lost in some lineages according to some literature (Chapco & Contreras, 2011; Nattier et al., 2011; Amorim et al., 2020).

“Gomphocerinae” is derived from the Greek word “gomphos” that meaning “inflated” and “ceros”, indicating that the distal end of the antennae of some male species has club shape. According to Yin’s perspective, he classified only those species with clubbed shaped antennae in the subfamily of Gomphocerinae (Yin, 1982). Based on morphological observations, the clubbed antennae are divided into two types: one with a distinctly inflated in the antennae end, such as the *Dasyhippus* Uvarov, 1930 and *Gomphocerus* Thunberg, 1815, and the other with a slightly inflated in the antennae end, such as *Myrmeleotettix* Bolívar, 1914 and *Aeropedellus* Hebard, 1935. However, most orthopterists believe that the shape of antennae within the Gomphocerinae is not uniform. In some genera (*e.g*., *Stenobothrus*), males and females may have different antennae. Moreover, morphological, karyology, comparative analysis of molecular data on both nuclear and mitochondrial genomes show that members of this subfamily have quite different antennae, from filiform to ensiform or clubbed (Sukhikh et al., 2019; Hawlitschek et al., 2022), this could be the result of convergent evolution.

As a major consumers of plants, grasshoppers play a crucial role in the functioning of global ecosystems, are an important component of food chains, and represent important agricultural pests (Song et al., 2018; Hawlitschek et al., 2017; Naz et al., 2020). Therefore, accurate identification of species is important for pest control, and requires traditional morphological methods and further verification from a molecular perspective. With the development of sequencing technology, molecular phylogenetic can use the differences in DNA sequences between species at the genetic level to further explore species identification and phylogenetic relationships.

Insect mitochondrial genomes comprise a double stranded circular DNA molecule structure with the size of 14 to 20 kb, including 13 protein-coding genes (PCGs), 22 transfer RNAs (tRNAs), 2 ribosomal RNA genes (12SrRNA, 16SrRNA) and one A+T control region (Boore, 1999; Cameron, 2014; Curole & Kocher, 1999). Because of strict matrilineal inheritance and highly conserved characteristics, mitochondrial genomes have become important for studying phylogeny and evolution (Yan et al., 2021; Lu, Huang & Deng, 2023; Zhang et al., 2023). Molecular phylogenetic analyses have examined the phylogenetic relationships of Gomphocerinae species within the subfamily and the family Acrididae (Zhang et al., 2013; Hawlitschek et al., 2022; Sukhikh et al., 2019), but so far it remains controversial (Bugrov et al., 2006; Chapco & Contreras, 2011; Nattier et al., 2011). In order to investigate the true phylogenetic relationships in Gomphocerinae species and the position of this subfamily within Acrididae, more powerful molecular markers are needed for further exploration.

In this research, complete mitogenomes of *Dasyhippus peipingensis*, *Myrmeleotettix palpalis*, and *Aeropedellus prominemarginis* were sequenced and analyzed. We compared the genomic organization and composition with other Gomphocerinae species and established its phylogenetic position in Acrididae using two different methods. We also constructed divergent time trees using BEAST to estimate the divergence times of these species in Acrididae. The results also provide contribute to further understanding in taxonomy and phylogeny evolution of the Acrididae.

## Materials and Methods

### Sample collection and DNA extraction

The samples used in this study are shown in Table 1. Specimens were preserved in 95% alcohol and then transferred to 4 °C for cryopreservation. We performed an accurate identification under stereomicroscope based on its morphological characteristics by Fauna Sinica (Yin & Xia, 2003). Using the Animal Tissues/Cells Genomic DNA Extraction Kit (Solarbio, Beijing, China) and according to the manufacturer’s instructions, total genomic DNA was extracted from the hind femora muscle. The quality of the total DNA was checked with a 1% agarose gel and the concentration was measured with a Nanodrop 2000 spectrophotometer. DNAs were stored at −20 °C for long term storage and further molecular analyses.

**Table 1:** Information on collecting samples.

Species	Collecting time	Collecting sites	Collector
*Dasyhippus peipingensis*	June 2009	Yuxian, Hebei	LI Xin-Jiang
*Myrmeleotettix palpalis*	August 2022	Chengde, Hebei	Xue Xiao-Bao
*Aeropedellus prominemarginis*	August 2008	Zhiduo, Qinghai	LI Xin-Jiang

### DNA sequencing and assembly

Complete mitochondrial genomes were sequenced using the Illumina Novaseq 6000 platform with 151 bp paired end reads at Personalbio, Shanghai, China. Using the invertebrate genetic code in Genious 8.1.3 (Kearse et al., 2012), by using the closely related known grasshoppers as reference sequences, complete mitochondrial genomes of *Dasyhippus peipingensis*, *Myrmeleotettix palpalis*, and *Aeropedellus prominemarginis* were assembled and aligned by using ClustalW (Larkin et al., 2007). Complete mitochondrial genome sequences were manually proofread in Genious 8.1.3 to check the accuracy of the assembly.

### Mitogenome assembly and annotation

The mitochondrial genome sequences were annotated using Genious 8.1.3 and online website GeSeq (https://chlorobox.mpimp-golm.mpg.de/geseq.html). The mitochondrial genome map was visualized by Proksee (https://proksee.ca/). The tRNA Scan-SE v. 1.21(Lowe & Eddy, 1997) was used to identify tRNAs and confirm their secondary structure, and the secondary structure was visualized using Adobe lllustrator 2020. The nucleotide composition, base composition skew, codon usage and relative synonymous codon usage (RSCU) of protein-coding genes were analyzed by PhyloSuite v1.2.2 (Zhang et al., 2019). The skew values were calculated using the formulae: GC skew = (G − C)/(G + C) and AT skew = (A − T)/(A + T) (Perna & Kocher, 1995). The “ggplot2” package in the RStudio was used to visualize relative synonymous codon usage (RSCU). Nucleotide diversity (Pi) and sliding window analysis (sliding window: 100 bp, step size: 25 bp) of 13 PCGs among 10 Gomphocerinae species was performed using DnaSP 5.0 (Librado & Rozas, 2009). Non-synonymous substitutions (Ka) and synonymous substitutions (Ks) were calculated for total PCGs by KaKs Calculator Toolbox 2.0 (Wang et al., 2009), and the evolutionary rate of PCGs was assessed by Ka/Ks value.

### Phylogenetic analyses and divergence time estimate

The complete mitochondrial genomes of 34 species from 13 subfamilies within family Acrididae were selected as ingroup, and seven species within Pamphagidae, Pyrgomorphoidea, and Tetrigoidea were chosen as outgroups (Table 2). All mitochondrial genomes (except the three sequenced in this study) were obtained from GenBank (accession numbers given in Table 2). The heterogeneity of nucleotide divergence of two matrixes were analyzed by AliGROOVE 1.5 (Kück et al., 2014). The nucleotide sequences of the 13 protein-coding genes were analyzed. Substitution saturation of PCGs based on Xia’s test implemented in DAMBE (Xia, 2017). The 13 PCGs were extracted by PhyloSuite v1.2.2 and aligned using MAFFT v7.313 (Katoh & Standley, 2013). Intergenic gaps were removed by Gblocks v 0.91b (Castresana, 2000), and the 13 protein-coding genes were concatenated in PhyloSuite v1.2.2.

**Table 2:** Summary of mitogenomes used in this study.

Family	Subfamily	Species	Accession no	References
Tetrigidae	Tetriginae	*Tetrix japonica*	NC_018543.1	Xiao et al. (2012)
		*Tetrix ruyuanensis*	NC_046412.1	Unpublished
Pamphagidae	Thrinchinae	*Humphaplotropis culaishanensis*	NC_023535.1	Li et al. (2016)
		*Filchnerella nigritibia*	MZ433420.1	Zheng et al. (2021)
Pyrgomorphidae	Pyrgomorphinae	*Atractomorpha sinensis*	NC_011824.1	Unpublished
		*Tagasta indica*	NC_045930.1	Unpublished
		*Mekongiella kingdoni*	NC_023921.1	Zhi et al. (2016)
Acrididae	Acridinae	*Acrida cinerea*	NC_014887.1	Liu & Huang (2010)
		*Acrida willemsei*	NC_011303.1	Fenn et al. (2008)
		*Phlaeoba tenebrosa*	NC_029150.1	Song et al. (2016a)
	Calliptaminae	*Calliptamus abbreviatus*	NC_030626.1	Unpublished
	Catantopinae	*Diabolocatantops pinguis*	NC_042904.1	Unpublished
		*Traulia szetschuanensis*	NC_013826.1	Unpublished
		*Xenocatantops brachycerus*	NC_021609.1	Unpublished
	Cyrtacanthacridinae	*Chondracris rosea*	NC_019993.1	Unpublished
	Eyprepocnemidinae	*Shirakiacris shirakii*	NC_021610.1	Unpublished
	Gomphocerinae	*Arcyptera coreana*	NC_013805.1	Unpublished
		*Arcyptera meridionalis*	NC_039962.1	Unpublished
		*Gomphocerippus rufus*	NC_014349.1	Sun, Zheng & Huang (2010)
		*Gomphocerus licenti*	NC_013847.1	Unpublished
		*Gomphocerus sibiricus*	NC_021103.1	Zhang et al. (2013)
		*Gomphocerus sibiricus tibetanus*	NC_015478.1	Yin et al. (2012)
		*Myrmeleotettix sp*.	MK903595.1	Unpublished
		*Orinhippus tibetanus*	NC_023467.1	Song et al. (2016b)
		*Pacris xizangensis*	NC_023919.1	Zhang et al. (2016)
		*Dasyhippus peipingensis*	OR066428	This study
		*Myrmeleotettix palpalis*	OR066427	This study
		*Aeropedellus prominemarginis*	OR061135	This study
	Hemiacridinae	*Hieroglyphus tonkinensis*	NC_030587.1	Unpublished
	Melanoplinae	*Melanoplus differentialis*	NC_057646.1	Unpublished
	Oedipodinae	*Aiolopus thalassinus*	NC_034674.1	Unpublished
		*Oedaleus decorus asiaticus*	NC_011115.1	Ma et al. (2009)
		*Ceracris fasciata fasciata*	NC_043956.1	Gao, Chen & Jiang (2017)
		*Bryodema luctuosum luctuosum*	HQ833839.1	Zhi et al. (2013)
		*Epacromius coerulipes*	NC_052732.1	Unpublished
		*Sphingonotus ningsianus*	NC_046563.1	Unpublished
		*Trilophidia annulata*	NC_027179.1	Unpublished
	Oxyinae	*Oxya chinensis*	NC_010219.1	Unpublished
	Spathosterninae	*Spathosternum prasiniferum prasiniferum*	NC_046532.1	Unpublished
	Conophyminae	*Conophymacris viridis*	NC_046528.1	Unpublished
	Dericorythinae	*Dericorys annulata*	NC_046555.1	Unpublished

The best-fit models of each gene were selected by ModelFinder (Lanfear et al., 2017), maximum likelihood (ML) and Bayesian inference (BI) were selected based on AICc, the results are presented in Tables S1 and S2. ML and BI analyses were performed in IQ-TREE (Nguyen et al., 2015) and MrBays v3.2.7 (Ronquist & Huelsenbeck, 2003), respectively. The ML phylogenetic analyses were using “Ultrafast” algorithm (bootstrap number = 5,000). In BI phylogenetic analyses, Markov chain Monte Carlo (MCMC) run for 10,000,000 generations, sampling trees every 10,000 generations with the 25% burn in. The data were imported into Tracer v.1.7.2 (Rambaut et al., 2018), ESS >200 proving that the data were converged. The consensus trees were displayed and managed visually by iTOL (https://itol.embl.de) (accessed February 2023).

Divergence time in Aricidiae was estimated using the 13 PCGs with relaxed molecular clock model in BEAST 1.10.4 (Drummond et al., 2012). Coalescent: Constant Size model was used for the prior tree, ModelFinder was used to find the best model GTR+G+F4. Divergence time tree nodes from Chang et al. (2020) were used for calibration (separation time 115 Mya for Tetrigoidea; 71 Mya for Chrotogonidae; 56 Mya for Pamphagidae; 35 Mya for Catantopinae; 33 Mya for Oedipodinae). The Markov chain was run 100,000,000 generations, sampling every 10,000 generations, 25% was burn in. The stability of the results was verified by Tracer v1.7.2 with most parameters having more than 200 effective sample size (ESS) values. ChiPlot (https://www.chiplot.online/tvbot.html) was used to visualize maximum clade credibility tree with 95% highest probability density (95% HPD).

## Results and discussion

### Genome content and organization

**Figure 1:**
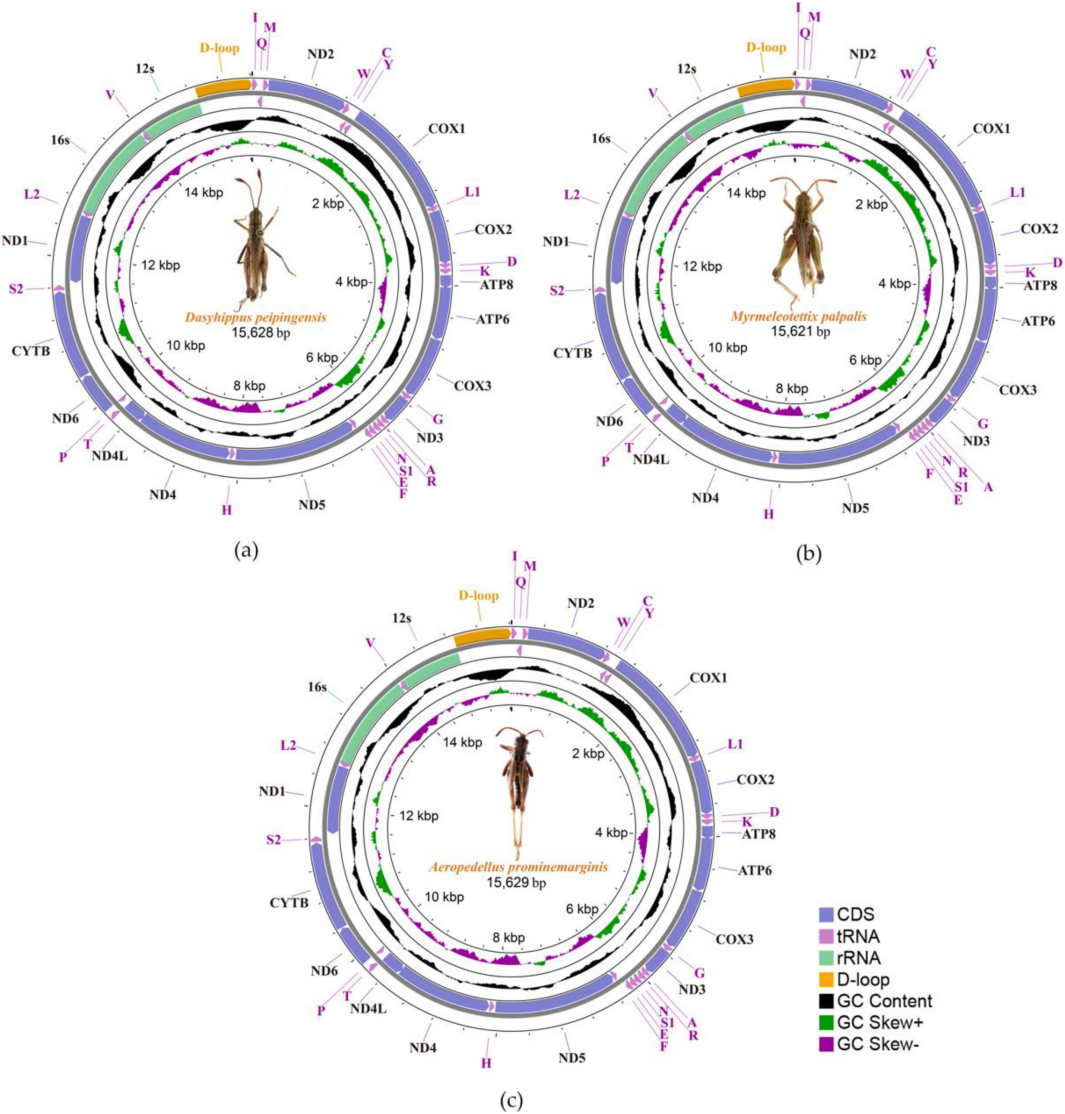
Three complete Mitochondrial genomes. (A) *Dasyhippus peipingensis*; (B) *Myrmeleotettix palpalis*; (C) *Aeropedellus prominemarginis*.

We sequenced and annotated the whole mitochondrial genomes which performed visual editing. The complete mitogenome sequence of *Dasyhippus peipingensis*, *Myrmeleotettix palpalis*, and *Aeropedellus prominemarginis* were 15,628, 15,621 and 15,629 bp, respectively. These mitogenomes showed typical insect mitogenome structure, which composed of circular double-stranded DNA molecules (Fig. 1). Each mitogenome includes 13 protein-coding genes (PCGs), 22 tRNA genes, two rRNA genes and an A+T rich region (control region). There were 23 genes (including nine PCGs and 14 tRNAs) are encoded on majority-strand (J-strand) and 14 genes (including four PCGs, eight tRNAs and two rRNAs) are transcribed from the minority-stand (N-strand) (Table 3).

**Table 3:** List of total size and intergenic nucleotides for mitochondrial genes with lengths of genes, anticodons of tRNAs and start/stop codons of protein-coding genes.

Gene	Coding strand	Nucleotide number	Size(bp)	Intergenic length	Anticodon	Initiation codon	Termination codon
tRNA-Ile	J	1-67/./.	67/./.	0/./.	GAT/./.		
tRNA-Gln	N	71-139/./.	69/./.	3/./.	TTG/./.		
tRNA-Met	J	143-211/142-210/.	69/./.	3/2/.	CAT/./.		
ND2	J	212-1234/211-1233/.	1023/./.	0/./.		ATG/./.	TAA/./.
tRNA-Trp	J	1233-1300/1232-1299/.	68/./.	-2/./.	TCA/./.		
tRNA-Cys	N	1293-1356/1292-1354/1293-1355	64/63/63	-8/./.	GCA/./.		
tRNA-Tyr	N	1369-1436/1367-1433/1368-1435	68/67/.	12/./.	GTA/./.		
COX1	J	1429-2973/1426-1970/1428-2972	1545/./.	-8/./.		ATC/./.	TAA/./.
tRNA-Leu(UUR)	J	2969-3034/2966-3031/2968-3033	66/./.	-5/./.	TAA/./.		
COX2	J	3037-3720/3033-3716/.	684/./.	2/1/3		ATG/./.	TAA/./.
tRNA-Asp	J	3719-3783/3715-3779/.	65/./.	-2/./.	GTC/./.		
tRNA-Lys	J	3786-3856/3782-3852/3786-3855	71/./70	2/./.	CTT/./.		
ATP8	J	3872-4033/3867-4028/3871-4032	162/./.	15/14/.		ATC/./.	TAA/./.
ATP6	J	4027-4704/4022-4699/4026-4703	678/./.	-7/./.		ATG/./.	TAA/./.
COX3	J	4709-5500/4704-5495/4708-5499	792/./.	4/./.		ATG/./.	TAA/./.
tRNA-Gly	J	5503-5569/5498-5563/5502-5568	67/66/.	2/./.	TCC/./.		
ND3	J	5570-5923/5564-5917/5569-5922	354/./.	0/./.		ATT/./.	TAA/./.
tRNA-Ala	J	5924-5989/5918-5983/5923-5988	66/./.	0/./.	TGC/./.		
tRNA-Arg	J	5989-6054/5987-6053/5992-6057	66/67/.	-1/3/3	TCG/./.		
tRNA-Asn	J	6057-6123/6056-6123/.	67/68/.	2/./1	GTT/./.		
tRNA-Ser(AGN)	J	6124-6190/./6128-6194	67/./.	0/./4	GCT/./.		
tRNA-Glu	J	6191-6256/./6195-6260	66/./.	0/./.	TTC/./.		
tRNA-Phe	N	6257-6320/./6261-6324	64/./.	0/./.	GAA/./.		
ND5	N	6322-8040/6321-8039/6325-8043	1719/./.	1/0/0		ATT/./.	TAA/./.
tRNA-His	N	8056-8122/8055-8120/8059-8125	67/66/.	15/./.	GTG/./.		
ND4	N	8127-9461/8125-9459/8130-9464	1335/./.	4/./.		ATG/./.	TAA/TAG/.
ND4L	N	9455-9748/9453-9746/9458-9751	294/./.	-7/./.		ATG/./.	TAA/./.
tRNA-Thr	J	9751-9819/9749-9817/9754-9822	69/./.	2/./.	TGT/./.		
tRNA-Pro	N	9820-9881/9818-9822/9823-9884	62/65/.	0/./.	TGG/./.		
ND6	J	9884-10405/9885-10406/9887-10408	522/./.	2/./.		ATG/./.	TAA/./.
cytb	J	10414-11550/10415-11554/10417-11553	1137/1140/.	8/./.		ATG/./.	TAA/./.
tRNA-Ser(UCN)	J	11562-11631/11563-11632/11565-11634	70/./.	11/8/.	TGA/./.		
ND1	N	11653-12597/11654-12598/11656-12600	945/./.	21/./.		ATA/./.	TAG/./.
tRNA-Leu(CUN)	N	12601-12667/12602-12667/12604-12670	67/66/.	3/./.	TAG/./.		
rrnL	N	12666-13981/12668-13979/12669-13987	1316/1312/1319	-2/0/.			
tRNA-Val	N	13981-14051/13980-14050/13987-14057	71/./.	-1/0/.	TAC/./.		
rrnS	N	14051-14894/14051-14893/14057-14899	844/843/843	-1/0/.			
D-loop	J	14894-15629/14894-15621/14900-15628	735/727/728	0/./.			

**Note:**

N and J indicate that the gene was located in the minor (N) and major (J) strand. “/.”: same as the one previous to it. The species are as follows: *Dasyhippus peipingensis*; *Myrmeleotettix palpalis*; *Aeropedellus prominemarginis*.

The nucleotide compositions of the three mitochondrial genomes revealed a distinct A/T bias: 75.7% (*Dasyhippus peipingensis*), 75.2% (*Myrmeleotettix palpalis*), and 75.0% (*Aeropedellus prominemarginis*). All mitochondrial genomes were positive for A+T skew and negative for GC skew (Table 4). The complete mitochondrial genomes and PCGs of three grasshopper species had A+T contents higher than 64% at different compositional sites and locations. The control regions (A+T-rich region) of the mitochondrial genomes were all located between tRNA-Ile and rrnS, with sizes of 729 bp (*Dasyhippus peipingensis*), 728 bp (*Myrmeleotettix palpalis*), and 735 bp (*Aeropedellus prominemarginis*), and the A+T content was >83%, which were also referred to as AT-rich regions. The structures and nucleotide compositions of the three species are generally consistent with the mitochondrial genome structure of the Acrididae (Zhang et al., 2023; Zheng et al., 2021), indicating that these mitochondrial genome’s structure is highly conserved (Wei et al., 2010).

**Table 4:** Nucleotide composition of three mitochondrial whole genomes.

Species	Regions	Size (bp)	T%	C%	A%	G%	A+T%	GC (%)	AT-skew	CG-skew
*Dasyhippus peipingensis*	Full genes	15,628	32.8	14.0	42.9	10.4	75.7	24.4	0.13	−0.15
	rRNAs genes	2,162	43.5	8.9	33.6	14.0	77.1	22.9	−0.13	0.22
	tRNAs genes	1,475	35.9	11.6	37.3	15.1	73.2	26.7	0.02	0.13
	A+T-rich region	729	37.2	9.3	46.1	6.9	83.3	16.2	0.02	−0.15
	PCGs	11,190	42.7	12.4	32.4	12.5	75.1	24.9	−0.14	0.00
	All codons									
	1st	3,730	36.0	11.7	33.3	19.0	69.3	30.7	−0.04	0.24
	2nd	3,730	45.9	19.8	20.1	14.1	66.0	33.9	−0.39	−0.17
	3rd	3,730	46.2	5.5	43.4	4.6	89.6	10.1	−0.03	−0.09
	Genes on J-strand									
	1st	2,299	29.7	13.9	37.0	19.4	66.7	33.3	0.11	0.17
	2nd	2,299	44.3	21.5	20.8	13.4	65.1	34.9	−0.36	−0.23
	3rd	2,299	36.2	8.0	53.5	2.3	89.7	10.3	0.19	−0.55
	Total	6,897	36.7	14.5	37.1	11.7	73.8	26.2	0.01	−0.11
	Genes on N-strand									
	1st	1,431	46.0	8.3	27.5	18.2	73.5	26.5	−0.25	0.37
	2nd	1,431	48.6	17.1	18.9	15.3	67.5	32.4	−0.44	−0.06
	3rd	1,431	62.3	1.5	27.9	8.3	90.2	9.8	−0.38	0.69
	Total	4,293	52.3	9.0	24.8	13.9	77.1	22.9	−0.36	0.22
*Myrmeleotettix palpalis*	Full genes	15,621	32.3	14.3	42.9	10.5	75.2	24.8	0.14	−0.15
	rRNAs genes	2,155	44.4	9.0	32.0	14.7	76.4	23.7	−0.16	0.24
	tRNAs genes	1,476	35.3	12.1	37.3	15.4	72.6	27.5	0.03	0.12
	A+T-rich region	728	39.4	9.1	45.3	6.2	84.7	15.3	0.07	−0.19
	PCGs	11,193	42.5	12.6	32.0	12.8	74.5	25.4	−0.14	0.01
	All codons									
	1st	3,731	35.9	11.9	32.9	19.3	68.8	31.2	−0.04	0.24
	2nd	3,731	45.6	20.2	19.8	14.3	65.8	34.5	−0.39	−0.17
	3rd	3,731	46.2	5.9	43.3	4.7	89.5	10.6	−0.03	−0.11
	Genes on J-strand									
	1st	2,300	29.3	14.0	36.9	19.8	66.2	33.8	0.11	0.17
	2nd	2,300	44.0	22.0	20.5	13.5	64.5	35.5	−0.37	−0.24
	3rd	2,300	36.2	8.2	53.2	2.4	89.4	10.6	0.19	−0.54
	Total	6,900	36.5	14.7	36.9	11.9	73.4	26.6	0.01	−0.11
	Genes on N-strand									
	1st	1,431	46.4	8.5	26.6	18.4	73.0	26.9	−0.27	0.37
	2nd	1,431	48.1	17.4	18.7	15.7	66.8	33.1	−0.44	−0.05
	3rd	1,431	62.2	2.1	27.3	8.5	89.5	10.6	−0.39	0.60
	Total	4,293	52.2	9.3	24.2	14.2	76.4	23.5	−0.37	0.22
*Aeropedellus prominemarginis*	Full genes	15,629	32.5	14.4	42.5	10.6	75.0	25.0	0.13	−0.15
	rRNAs genes	2,160	42.8	9.4	32.9	14.9	75.7	24.3	−0.13	0.23
	tRNAs genes	1,476	35.4	12.0	37.3	15.3	72.7	27.3	0.03	0.12
	A+T-rich region	735	37.8	9.4	46.5	6.3	84.3	15.7	0.10	−0.20
	PCGs	11,190	42.3	12.7	32.1	12.9	74.4	25.6	−0.14	0.01
	All codons									
	1st	3,730	35.6	12.0	32.8	19.6	68.4	31.6	−0.04	0.24
	2nd	3,730	45.8	19.9	20.0	14.2	65.8	34.1	−0.39	−0.17
	3rd	3,730	45.5	5.6	43.5	4.8	89.0	10.4	−0.02	−0.08
	Genes on J-strand									
	1st	2,299	29.4	14.3	36.2	20.1	65.6	34.4	0.10	0.17
	2nd	2,299	44.2	21.6	20.7	13.5	64.9	35.1	−0.36	−0.23
	3rd	2,299	35.3	9.0	52.7	3.0	88.0	12.0	0.2	−0.502
	Total	6,897	36.3	15.0	36.5	12.2	72.8	27.2	0.00	−0.1
	Genes on N-strand									
	1st	1,431	45.6	8.4	27.3	18.7	72.9	27.1	−0.25	0.38
	2nd	1,431	48.4	17.2	18.9	15.4	67.3	32.6	−0.44	−0.05
	3rd	1,431	61.9	1.7	28.8	7.6	90.7	9.3	−0.37	0.64
	Total	4,293	52.0	9.1	25.0	13.9	77.0	23.0	−0.35	0.211

The total lengths of the 13 PCGs of *Dasyhippus peipingensis*, *Myrmeleotettix palpalis* and *Aeropedellus prominemarginis* are 11,190, 11,193 and 11,190 bp, respectively (Table 4), accounting for 71.6%, 71.7%, and 71.6% of the whole mitochondrial genome, respectively. The size of the PCGs ranges from 162 bp (ATP8) to 1,719 bp (ND5). Among the 13 PCGs, 9 PCGs (ATP6, ATP8, COX1, COX2, COX3, CYTB, ND2, ND3, and ND6) are encoded on the J-strand, while 4 PCGs (ND1, ND4, ND4L, and ND5) are encoded on the N-strand. The third codon position has the highest A+T content, while the second codon position has the lowest A+T content. All the initiation codons in the mitogenomes of the three species were ATN, with ATG being the most frequently used, termination codons were TAN, and TAA was the most frequently used termination codon.

**Figure 2:**
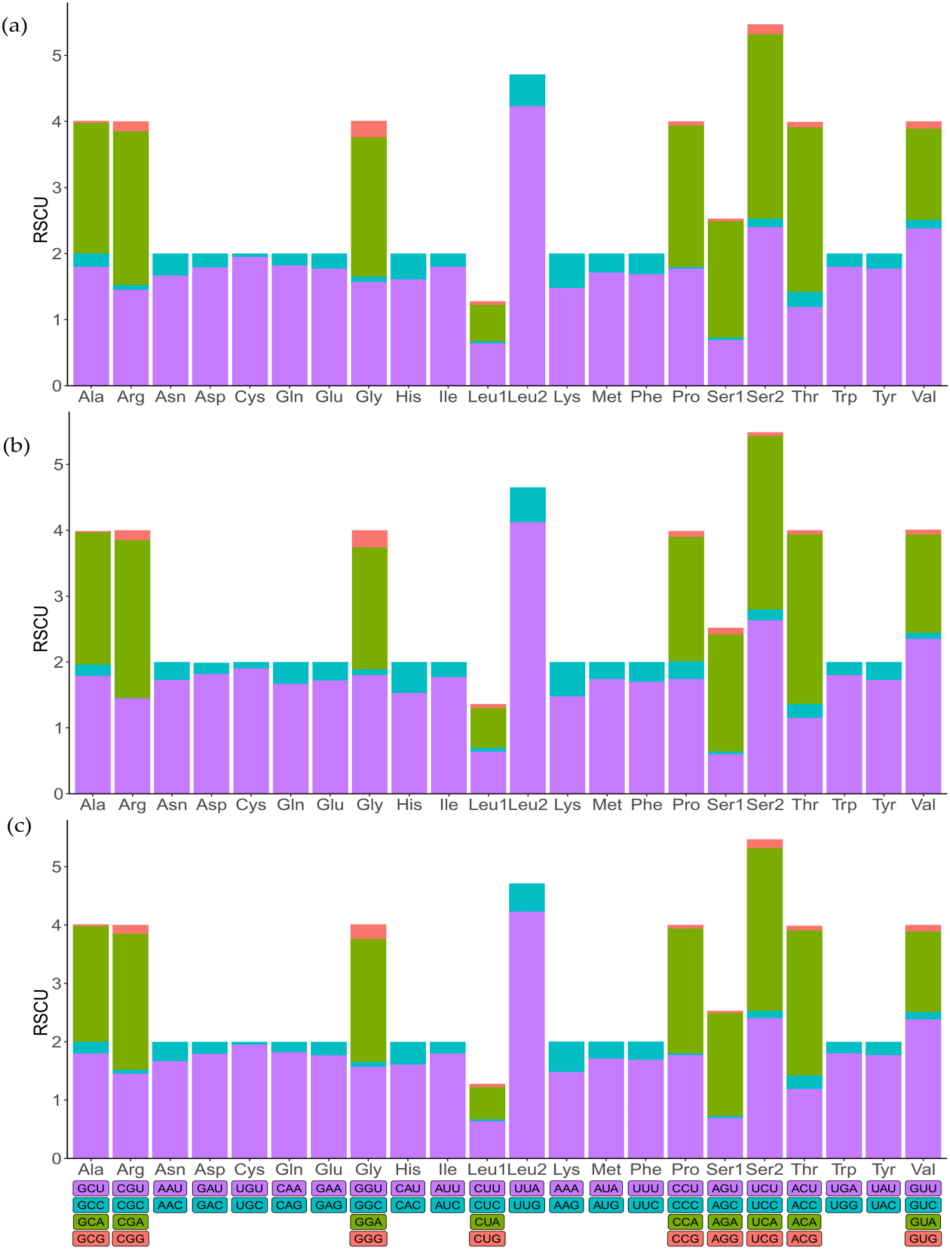
The relative synonymous codon usage (RSCU) in mitochondrial genomes. (A) *Dasyhippus peipingensis*; (B) *Myrmeleotettix palpalis*; (C) *Aeropedellus prominemarginis*.

To indicate the frequency of codon usage, the relative synonymous codon usage (RSCU) values of the three mitochondrial genomes were visualized (Fig. 2; Table S3). Comparative analysis showed that the synonymous codon preferences were highly conserved among three mitochondrial genomes. The most frequently used codons are TTT, TTA, ATT, and ATA, therefore, Phe, Leu (UUR), IIe, and Met are most frequently used amino acids, accounting for 7.86%, 9.67%, 9.31%, and 5.81% of total, respectively. In addition, RSCU analysis also showed a bias towards using more A/T at the third codon position rather than G/C. Similarly, the frequency of codon usage indicates the preference of nucleotide A/T in three species.

**Figure 3:**
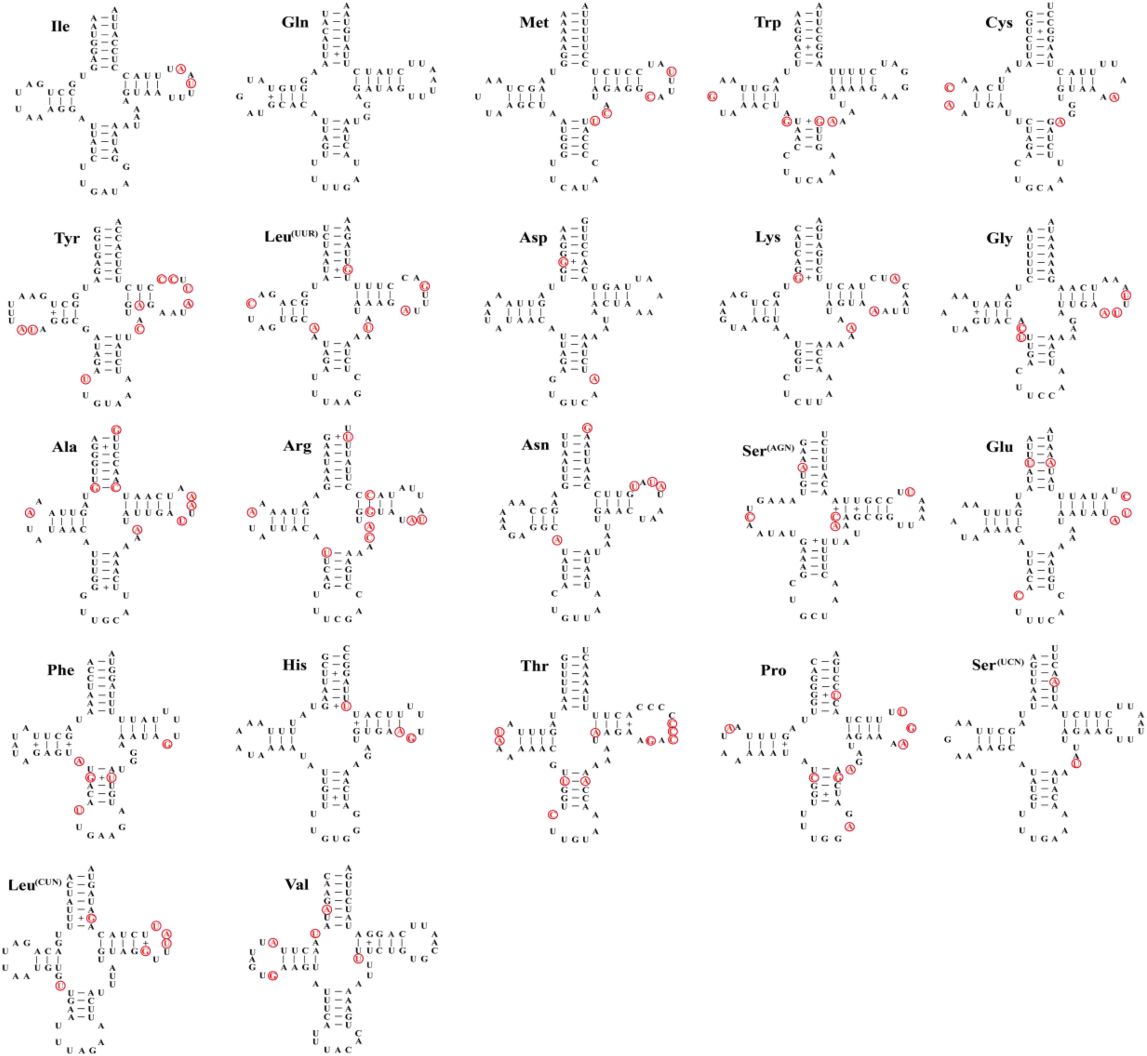
Twenty-two tRNA secondary structures identified in the mitochondrial genome of the *Aeropedellus prominemarginis*. Base pairing and mismatches indicated by (−) and (+), respectively. The variable sites in the mitochondrial genomes of the other two locust species are represented by red circles.

As other insects, the mitochondrial genome of three species contain 22 tRNA genes with lengths ranging from 62–71 bp (Table 3), the total length of tRNAs is ranging from 1,475 bp (*Dasyhippus peipingensis*) to 1,476 bp (*Myrmeleotettix palpalis* and *Aeropedellus prominemarginis*). The A+T content of tRNAs is 73.2%, 72.6%, and 72.7% for three mitochondrial genomes, with positive AT skew and GC skew. Most tRNAs could be folded into the typical clover leaf secondary structure, except that tRNA-Ser (AGN) lacked a dihydrouridine (DHU) arm and formed a simple loop (Fig. 3). The secondary structure of tRNAs is usually conserved in the amino acid acceptor arm and anticodon loop, while DHU and TψC are more variable. In addition to the classic base pairs A-U and C-G, there are also noncanonical base pairings (G-U and A-C) and mismatched base pairs (A-A and A-G) distributed throughout the tRNA arms, with G-U noncanonical base pairs being the most abundant.

The two ribosomal RNA genes are encoded on the N-strand among three grasshoppers (Table 3), rrnL is located between tRNA-Leu (CUN) and tRNA-Val, while rrnS is flanked by tRNA-Val and A+T rich regions. The rrnL of *Dasyhippus peipingensis*, *Myrmeleotettix palpalis* and *Aeropedellus prominemarginis* are 1,319, 1,316 and 1,312 bp in length, contains the A+T content ranging from 77% to 78%. The rrnS is 843bp in *Dasyhippus peipingensis* and *Myrmeleotettix palpalis*, 844 bp in *Aeropedellus prominemarginis*, with AT content ranging from 73.6% to 75.5%. Therefore, there were no significant differences in rRNAs among three species. Both rrnL and rrnS exhibit negative AT-skew and positive GC-skew in three mitogenomes.

**Figure 4:**
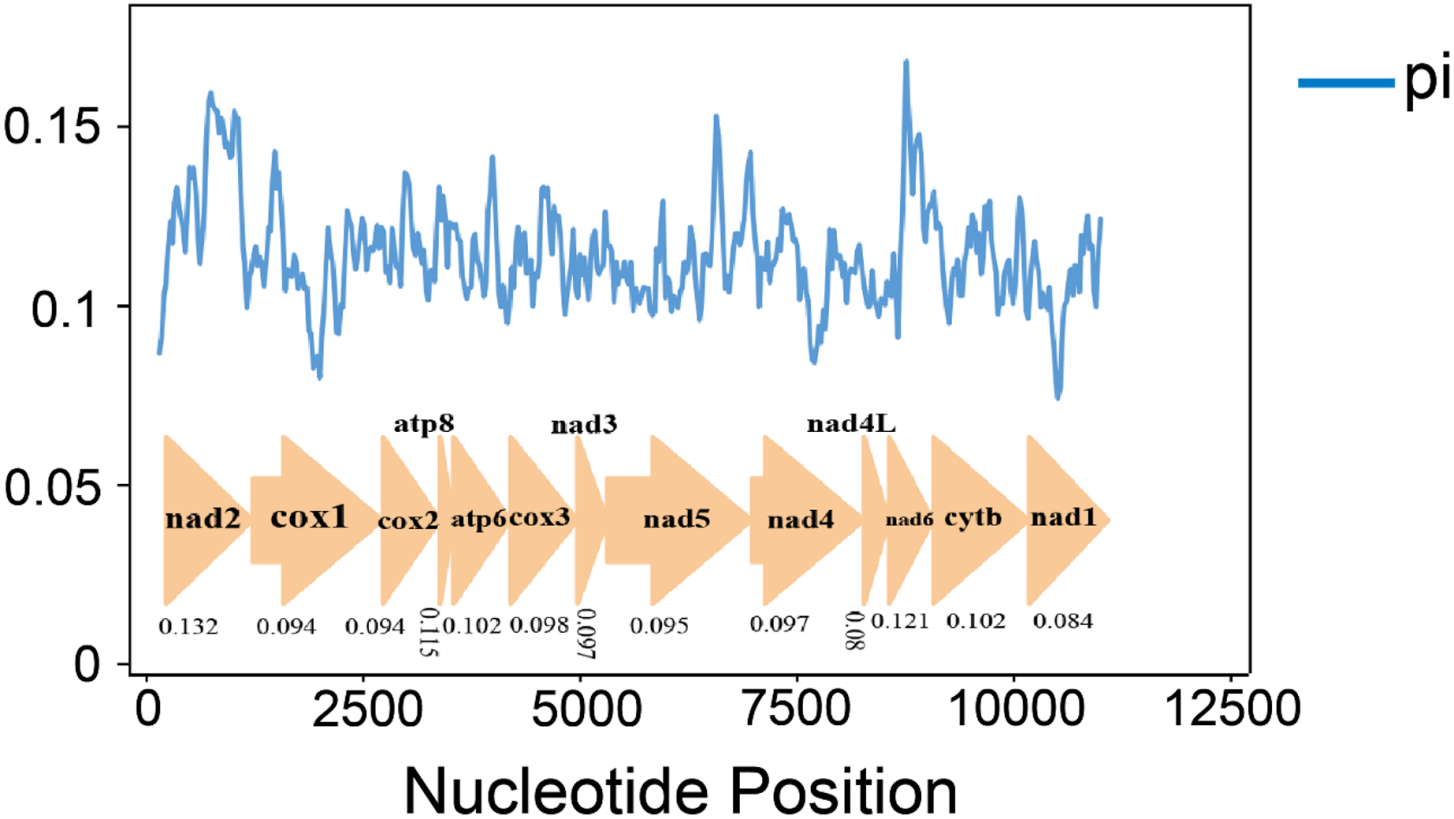
10 gomphocerine species PCGs nucleotide diversity. The blue curve represents the value of nucleotide diversity (Pi), and the average value of each gene is displayed below the gene name.

Nucleotide diversity analysis can identify regions with large nucleotide divergence, which is useful for designing species-specific markers in groups within taxa where morphological identification is difficult and taxonomic boundaries are blurred (Ma et al., 2019; Xie et al., 2011; Yuan et al., 2022). The nucleotide diversity (Pi values) of 10 species was analyzed by sliding window analysis (Fig. 4). In the mitochondrial genomes of the 10 gomphocerine species, nucleotide diversity is highly variable. Nucleotide diversity ranges from 0.08 to 0.132, with higher nucleotide diversity in genes ND2, ND6, and ATP8, which are 0.132, 0.121, and 0.115, respectively. In contrast, the nucleotide diversity of COX1, ND1, and ND4L is lower, with 0.094, 0.084, and 0.080, respectively. This indicates that COX1, ND1, and ND4L are relatively conserved genes.

**Figure 5:**
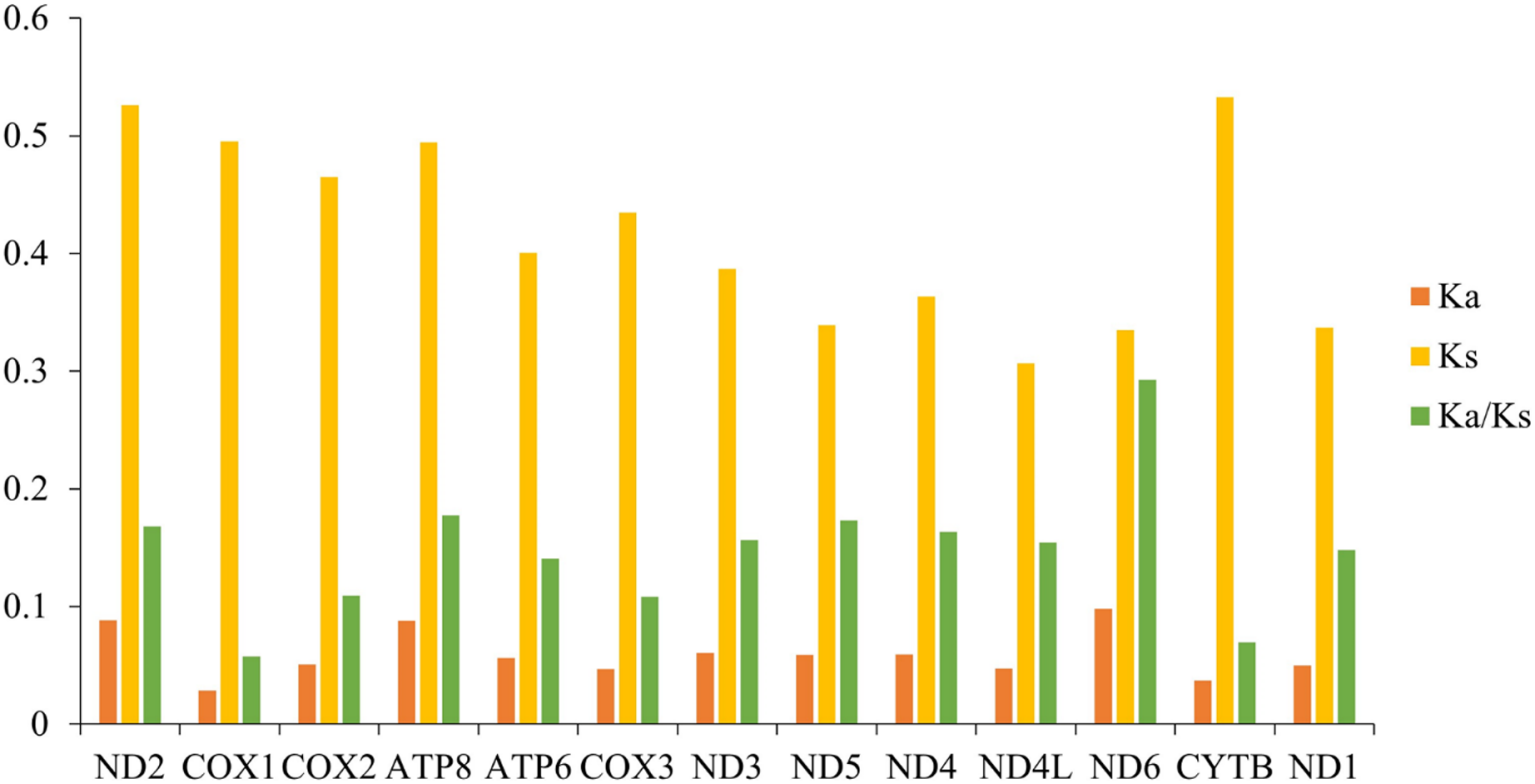
Synonymous substitution rate (Ka) and non-synonymous substitution rate (Ks) as well as the Ka/Ks ratio of PCGs in 10 grasshoppers.

Ka/Ks indicates the ratio between the non-synonymous substitution rate (Ka) and the synonymous substitution rate (Ks) of two protein-coding genes, which can be used as an important marker to estimate the evolutionary rate. We calculated the Ka/Ks values of the mitochondrial genomes among 10 species of gomphocerine (Fig. 5; Table S4). The Ka/Ks values of all PCGs were less than one, indicating that these genes evolved under purifying selection and were evolutionarily conserved in the mitochondrial genome. ND6 had the highest Ka/Ks value, followed by ATP8 and ND5 and COX1 had minimum Ka/Ks value (Ka/Ks = 0.058) and low evolutionary rate, indicating that the COX1 gene had strong purifying selection and evolutionary conservation, which could be used as an important marker to identify relatedness among species, therefore, a partial fragment of COX1 is often used as DNA barcodes for inferring species phylogenetic relationships (Hebert, Ratnasingham & deWaard, 2003). In contrast, ND6 had the highest Ka/Ks value (Ka/Ks = 0.293), showing a faster evolutionary rate with less selection pressure in PCGs, which undergone relatively weak purifying selection. It can be used to assess intraspecific relationships and is more suitable as a potential molecular marker in population genetics (Yuan et al., 2021; Pu et al., 2022; Chen et al., 2021).

### Phylogenetic analysis

**Figure 6:**
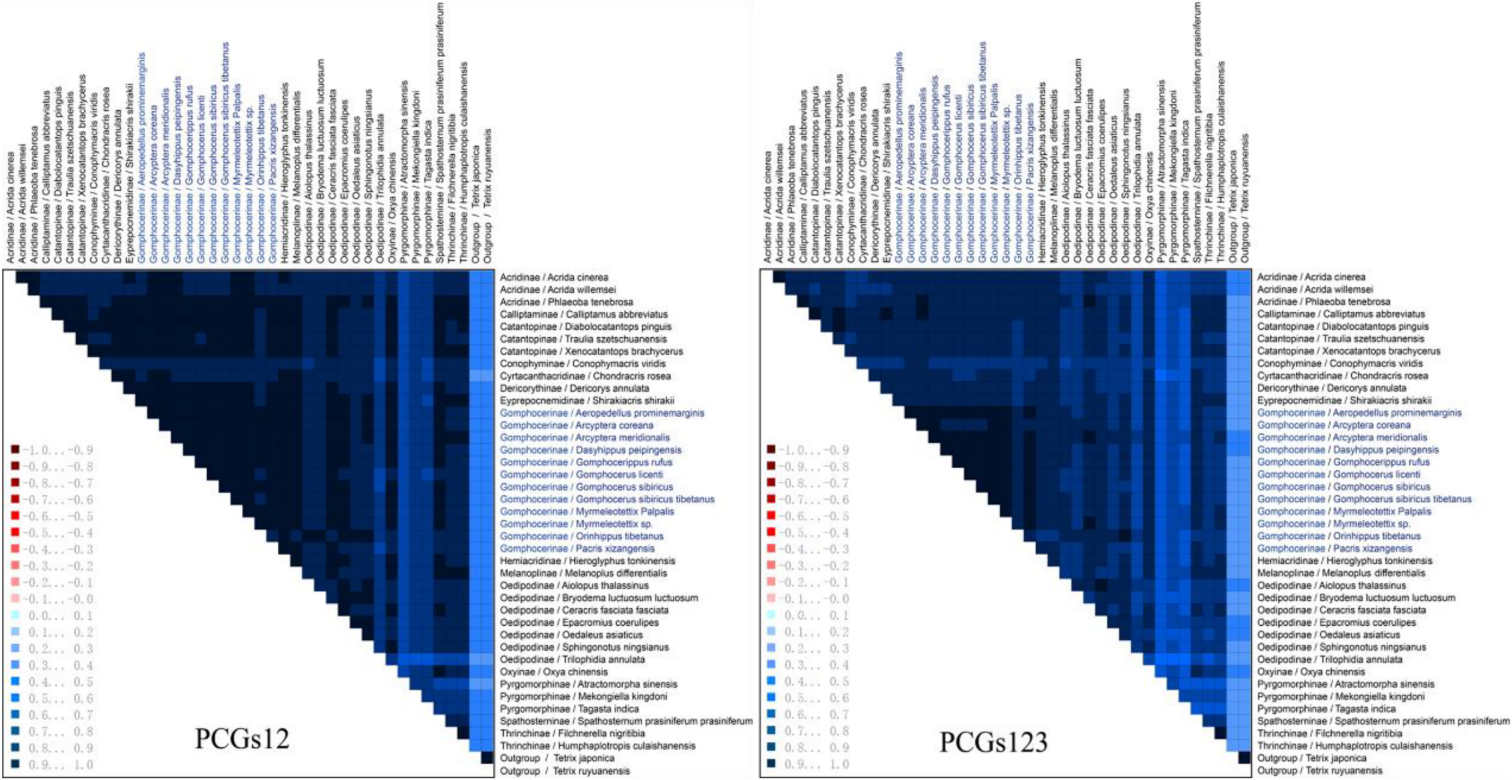
Mitochondrial genome sequences heterogeneity differences in the matrix PCG123 and PCG12. The average similarity score between the sequences is represented by a colored square. The AliGROOVE score ranges from −1 to +1 (the red part indicates significant heterogeneity, and the dark part indicates significant homogeneity).

The heterogeneity sequence divergence of the two matrixes PCG123 and PCG12 was assessed (Fig. 6), both indicated that the mitochondrial genomes of gomphocerine species showed lower heterogeneity (the similar scores for pairwise sequence comparisons were the lowest). Furthermore, among the species in Gomphocerinae, *Orinhippus tibetanus* shows higher heterogeneity than others. Substitution saturation of PCGs of 41 sequences were tested, and Xia’s analysis showed Iss < Iss.c and *p* < 0.05, revealed that base substitutions had not saturated and phylogenetic analysis could be performed.

**Figure 7:**
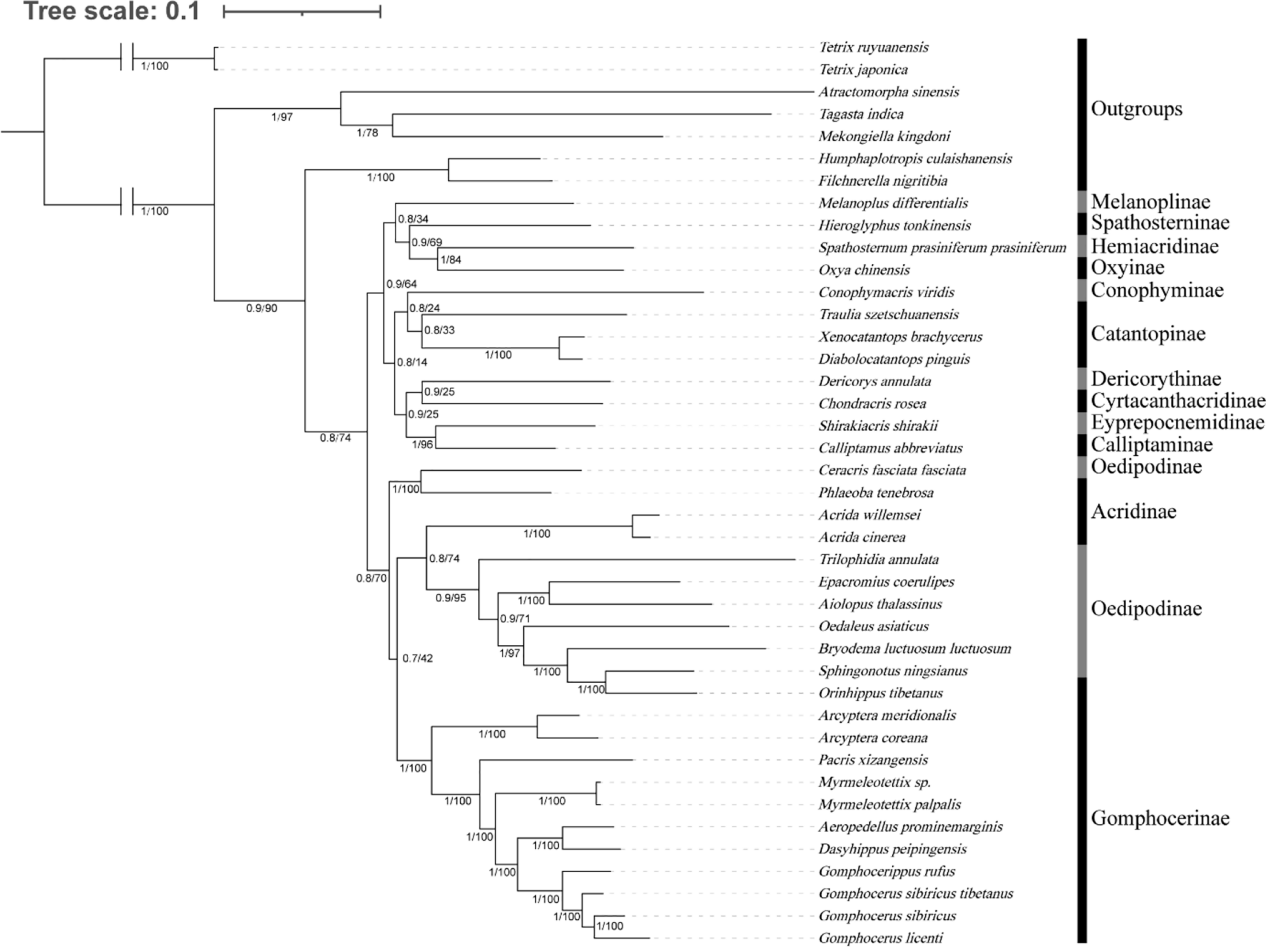
Phylogenetic evolution inferred from the BI and ML of the PCG123 matrix. The numbers on the branches represent the posterior probability (PP)/bootstrap value (BS).

We investigated the phylogenetic position of species among Gomphocerinae within Acrididae (Fig. 7). Tree topologies were consistent from both BI and ML analysis with high bootstrap values (BS) and bayesian posterior probability values (PP) in most clades. In Gomphocerinae, except for *Orinhippus tibetanus*, all other species showed significant monophyly (PP = 100, BS = 1). *Orinhippus tibetanus* and the species of Oedipodinae are recovered as sister groups, which is greatly supported (PPs = 1, BSs = 100). The results further support the reclassification of *Orinhippus tibetanus* as a member of the Oedipodinae using molecular systematics by Gao, Chen & Jiang (2017). In addition, by means of species descriptions of the genus and type species of the *Orinhippus*, previous research has classified the genus as a member of Locustinae (=Oedipodinae) (Uvarov, 1921; Bei-Bienko & Mishchenko, 1951), our results corroborate this ancient view from a molecular point of view. Therefore, we believe that antennal structure is of lesser importance as a higher order taxonomic character for grasshoppers. Moreover, this study clarifies the phylogenetic status of the genus *Aeropedellus* in Gomphocerinae, which is recovered a sister group with the genus *Dasyhippus*.

**Figure 8:**
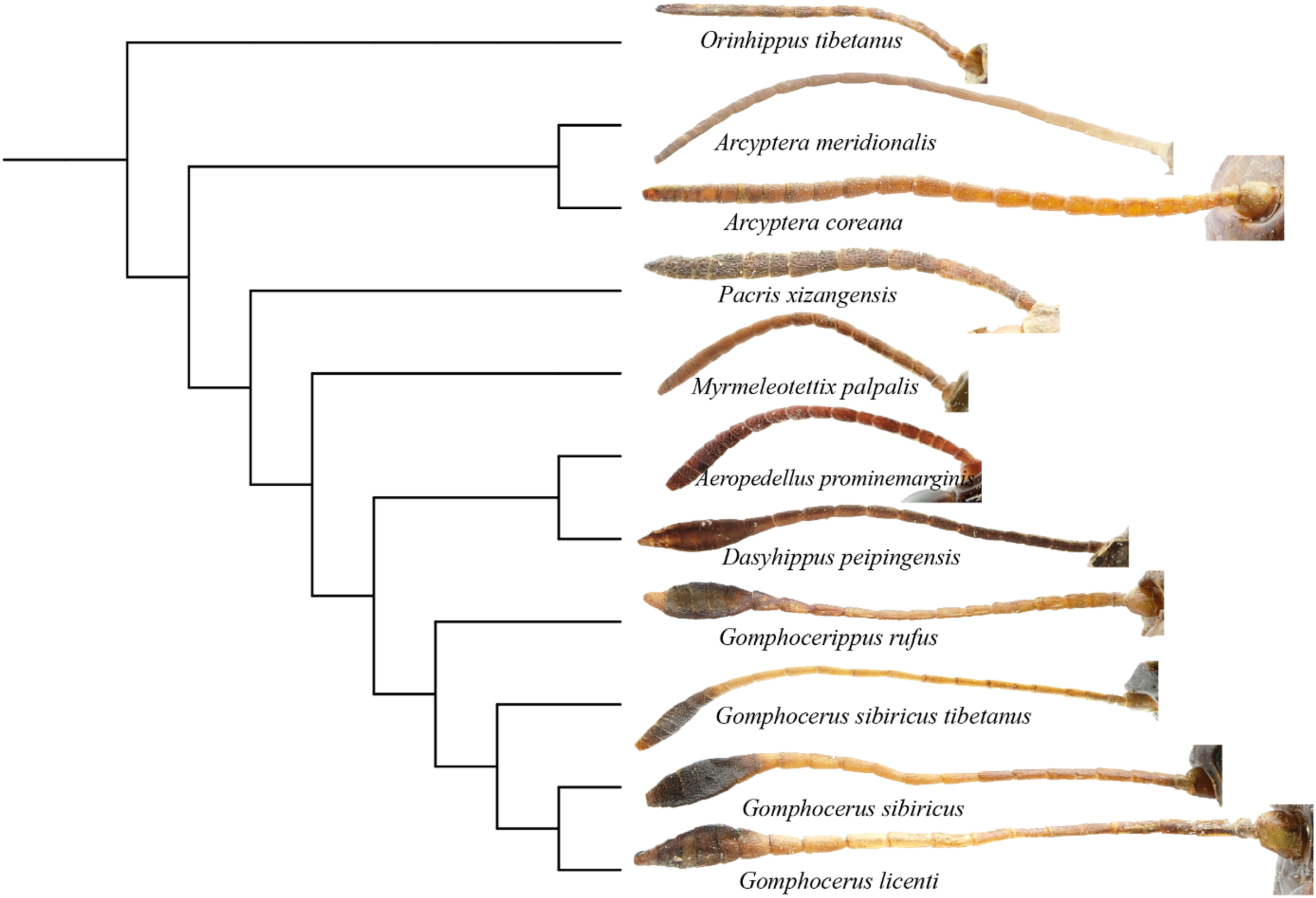
Phylogenetic evolution of antennae in Gomphocerinae. The topology is as shown in Fig. 7.

Phylogenetic analysis showed that the filiform antennae tend to be ancestral, while the condensed and expanded antennal flagellum which ends gradually to form a clubbed shape, appears to be more evolutionarily advanced (Fig. 8). This may first occur in micro-club-shape antennae of plateau species *Pacris xizangensis*, and the subsequently differentiated genera *Myrmeleotettix* and *Aeropedellus* show similar antennal morphology, in which the male antennal ends are slightly expanded at 5–7 segments, forming antennal ends that are twice as wide as long. Starting from *Dasyhippus*, the male antennal ends are very expanded at 7-8 segments, forming antennal ends that are significantly wider than long. Similar antennal morphology occurs in *Gomphocerus* and *Gomphocerippus*. The results indicated a possible antennal evolutionary trend in Acrididae, in which the filiform antennae are more ancient and gradually evolved into clubbed shape antennae.

Convergent evolution refers to independent lineages evolving similar phenotypes under similar selective pressures (Fraser & Whiting, 2019), but the phenomenon of convergent evolution is not easy to identify in evolution. Both *Orinhippus tibetanus* and *Pacris xizangensis* are distributed in Tibet, with very similar altitudes and environmental factors in their habitat, both species are classified in Gomphocerinae. However, based on phylogenetic analysis using mitochondrial genomes, *Orinhippus tibetanus* and *Pacris xizangensis* are on two independent clades. This study speculated that *Orinhippus tibetanus* and *Pacris xizangensis* may have been subjected to similar environmental selection pressures that formed similar antennal morphology convergently.

The phylogenetic relationships among Melanoplinae, Catantopinae, and Oxyinae are unclear in this tree, which may be the result of incomplete sampling. However, since they are not the focus of this research, no further discussion has been conducted. The low confidence level may be due to insufficient sampling, and in further studies, a wide range of sampling and multiple methods may be used to explore the phylogenetic relationships of these subfamily units in Acrididae. Further understanding of the triggering factors for evolution and the convergence of ecological forms in entire tree of life may help clarify genomic constraints and historical contingencies that have led to convergent evolution.

### Divergence time estimation

**Figure 9:**
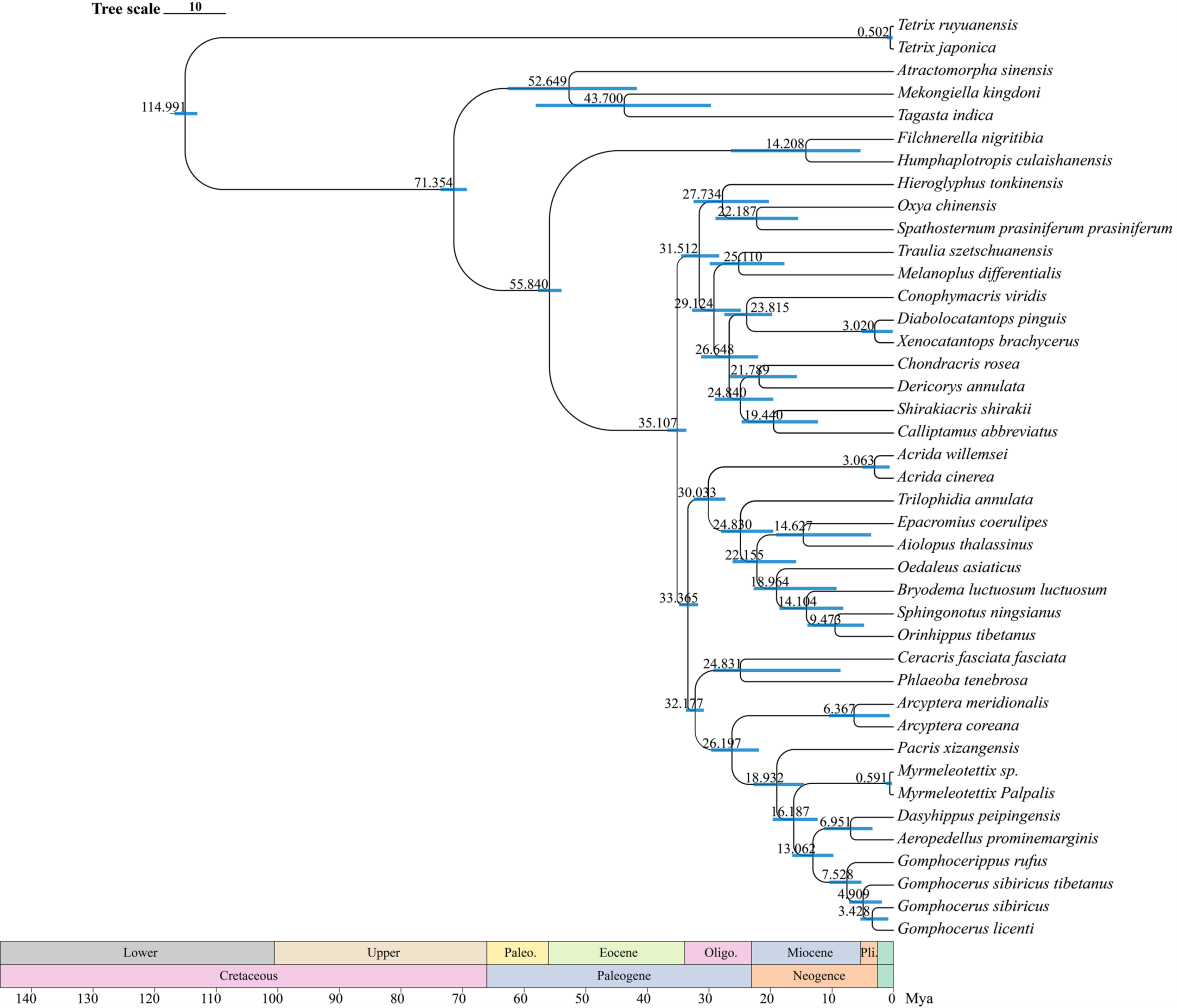
Divergence time tree. The blue bars represent the 95% confidence intervals of the estimated time, and the numbers on the nodes indicate the mean divergence time.

The divergence time estimates (95% HPD) of each species based on the topology recovered from BEAST analysis were exhibited in Fig. 9. The divergence time tree indicated that *Orinhippus* diverged first, early in the Oligocene 33 Mya (32–35 Mya, 95% HPD), while the divergence events of the other clubbed antennae species occurred in late Miocene 19 Mya (15–23 Mya, 95% HPD). The divergence between micro-clubbed-shape and clubbed-shape antennae occurred between *Aeropedellus* and *Dasyhippus*, and before divergence of *Gomphocerus* and *Gomphocerippus*, which was about 8–11 Mya.

According to the BEAST analysis, the formation of club shape antennae occurred approximately 18 Mya in *Pacris*, after which there were two independent evolutionary events, resulting in extreme enlargement of the antennal ends (*Gomphocerus* and *Dasyhippus*) and slight enlargement (*Myrmeleotettix* and *Aeropedellus*). The clubbed shape antennae are an important taxonomic characteristic at the family level, which is occurred only in species of the Gomphocerinae (Yin, 1982; Yin & Xia, 2003). However, the divergence times indicate that the earliest clade of the gomphocerine grasshoppers, *Pacris xizangensis*, diverged at approximately 18 Mya (15–23 Mya, 95% HPD). Compared with the divergence times of subfamilies such as Oedipodinae, Acridinae, and Melanoplinae, the clubbed antennae grasshoppers have a relatively brief divergence history, may not to reach the family category, which confirms the current classification system that places them in Gomphocerinae under the tribe Gomphocerini (Otte, 1981). The results of the divergence time estimates reconfirmed that antennal morphology should be given less weight as a taxonomic character for grasshoppers higher-level classification. It also suggests that species within the subfamily Gomphocerinae are not monophyletic.

Limitations in selecting gene fragments, lateral gene transfer during early evolution, or ancestral polymorphisms may result in phylogenetic incongruence between morphological and molecular data during speciation events. Feng et al. (2022) suggested that constructing a species relationship tree based on only partial genes and phenotypes may not be reliable, and genome-wide data is the gold standard for reconstructing the evolutionary history of species. However, the genome of grasshoppers is significantly larger than other insects (Alfsnes, Leinaas & Hessen, 2017; Husemann et al., 2020), whole genome sequencing is expensive and it is difficult to analyze the data. Therefore, it is often more practical to select more conservative molecular markers to explore the true evolutionary relationships among species in Acrididae. In addition, incomplete lineage sorting and convergent evolution may also cause contradictions between morphology and molecular data. The taxonomic category of clubbed antennae grasshoppers in Acrididae requires deeper investigation using larger scale sampling.

## Conclusions

The mitochondrial genomes of three Gomphocerinae species, *Dasyhippus peipingensis*, *Myrmeleotettix palpalis* and *Aeropedellus prominemarginis* were sequenced, annotated, and analyzed. The results demonstrated that size and structure of the mitochondrial genomes in the three species were conservative and identical to others in Acrididae. The nucleotide composition of three species showed a strong AT bias in mitochondrial genome. The codon usage of protein-coding genes was highly conserved, except for tRNA-Ser (AGN), which lacks a dihydrouridine (DHU) arm, all other tRNAs could fold into a typical cloverleaf structure. There was no significant difference in the size of rRNAs among three species. The Ka/Ks values of all PCGs were <1, indicating that these genes evolved under purifying selection.

This study used complete mitochondrial genomes to explore the phylogenetic relationships among several grasshoppers within Gomphocerinae and determined the phylogenetic status of the genus *Aeropedellus*. The results provide new and important information about the classification of Gomphocerinae. In Acrididae, differences in antennal shape should be given less weight as a taxonomic character for higher-level classification. To deeply explore the phylogenetic relationships among grasshoppers, increased sampling of taxa and selection of multiple genes is needed to reconstruct more comprehensive phylogenetic relationships.

## Supplemental Information

10.7717/peerj.16550/supp-1 Supplemental Information 1 The best-fit replacement models for the Maximum Likelihood (ML).

10.7717/peerj.16550/supp-2 Supplemental Information 2 The best-fit replacement models for the Bayesian Inference (BI).

10.7717/peerj.16550/supp-3 Supplemental Information 3 Codon number and RSCU in the three mitochondrial PCGs.

10.7717/peerj.16550/supp-4 Supplemental Information 4 The evolutionary rate of each PCGs in the mitogenomes of 10 gomphocerine species.

10.7717/peerj.16550/supp-5 Supplemental Information 5 41 complete and aligned mitochondrial PCG123 sequence datasets of grasshoppers.

10.7717/peerj.16550/supp-6 Supplemental Information 6 Mitochondrial gene data *Myrmeleotettix palpalis* with identification number OR066427.

10.7717/peerj.16550/supp-7 Supplemental Information 7 Mitochondrial gene data *Dasyhippus peipingensis* with identification number OR066428.

10.7717/peerj.16550/supp-8 Supplemental Information 8 Mitochondrial gene data *Aeropedellus prominemarginis* with identification number OR061135.

## Additional Information and Declarations

### DNA Deposition

The following information was supplied regarding the deposition of DNA sequences: The sequences are available at GenBank: OR066428, OR066427, OR061135. The data will be included in the Supplemental Files due to its unavailability for public disclosure. If the publication is completed, the data will be made publicly available.

### Data Availability

The following information was supplied regarding data availability: The raw data are available in the Supplemental Files.
